# Ingleby Lectures: Lecture III

**Published:** 1891-04-25

**Authors:** 


### SURGERY.

### INGLEBY LECTURES.—Lecture III.

In his third lecture Mr. Jordan Lloyd considered first

Inflammatory Diseases of Bones.

(1) *Acute Periostitis.*—The chief causes of this condition are (1) cold, (2) traumatism. It mostly occurs in the shaft of the tibia. Its diagnosis is often delayed, and it is often at first mistaken for rheumatism. There are many cases on record where a supposed case of rheumatism has been followed by necrosis, the mistaken diagnosis being thus forcibly demonstrated. In diagnosing this condition note whether heat and tenderness are present. Mark the situation of the disease, and, if still in doubt, make an exploratory incision. Acute periostitis is often followed by necrosis, and frequently extends into neighbouring joints, especially into those of the hip and ankle. The removal of the sequestrum in a case of necrosis should not be attempted too early, for in most cases, although by waiting the bone may become almost altogether devoid of periosteum, yet future repair will be well marked.

(2) *Bone Abscess.*—This condition is often overlooked. When diagnosed its efficient treatment is very satisfactory. It most commonly occurs at the upper and lower ends of the tibia. It is apt to extend into and cause destruction of the neighbouring joints. The chief symptoms are (*a*) paroxysmal localised pain; (*b*) circumscribed exquisite tenderness; (*c*) the chronicity of the disease. The best treatment is to trephine the bone over the abscess. A gouge will do very well for the purpose.

(3) *Epiphysitis.*—In this condition inflammation occurs on either side of the articular cartilage. It mostly occurs in children under one year of age and chiefly affects the knee and elbow joints.

The chief symptoms are (*a*) an annular swelling near the bone ends, acute pain; (*b*) increased heat over the part; (*c*) there is usually joint rigidity, but no effusion; (*d*) only one bone is usually affected at a time. The most frequent cause of this complaint is syphilis and hence it is wise always in the first place to try a mercurial course of treatment, and this is often very successful.

Dislocations.

Regarding dislocations, Mr. Lloyd stated that these injuries are rare in children because the bones are so soft and so are more apt to break than to become dislocated.

(1) *Pulled Elbow.*—This is a very common condition in children. The symptoms are a little pronation of the fore-arm tenderness over the elbow-joint, and yet there is no appreciable difference on examining the two limbs. The cause of this condition is a half unbuttoning of the head of the radius and the articular ligament. In treating it you flex and then supinate the limb, when a click will be heard and reduction has taken place.

(2) *Dislocation of the Head of the Radius Forwards.*—Mr. Lloyd read the notes of a case of the above injury which was characterised by the following symptoms: (*a*) a swelling in the triangular space on the front of the elbow; (*b*) a flattening of the forearm over the supinator brevis muscle. The reduction of the dislocation was easy, but could not be maintained; however, as it only checked somewhat the movement of flexion it was not regarded as a very serious condition although incurable.

(3) *Dislocation of the First Phalanx of the Thumb.*—Notes of a case of this kind were also read in which the flexor brevis pollicis muscle had to be divided before reduction could be effected.

Inflammation of Joints.

(1) *Acute Arthritis of Infants.*—This disease occurs generally in the first year of life, chiefly in the hip and in the knee. It begins within the joint, suppuration occurs early, and it is mostly fatal. It is frequently a result of syphilis, so that a mercurial course should be tried. If recovery should occur anchylosis is not essential.

(2) *Dr. Koch's Discovery.*—Mr. Lloyd said that he had now had nearly three months' personal experience of Dr. Koch's lymph. He had given it in even larger doses than recommended, and he did not hesitate to say that, in his hands, in the treatment of chronic surgical diseases of all kinds, it had been perfectly useless. Moreover, it could not be depended upon as a diagnostic agent, for in certain cases quite free from all tubercular taint he had got marked reactions after using the lymph. In fact, as a curative agent, the lymph was not worth the stamps required to bring it over from Berlin.

(3) *Hip-Joint Disease.*—The early signs of this disease are slight flexion and impaired function. In diagnosing a case of doubtful hip disease Thomas's method of examination should be employed, viz., bend the sound thigh on to the abdomen; if you can now depress the other thigh to the table there is no disease.

*Treatment.*—Mr. Lloyd advocates the plan of (*a*) applying a weight and pulley to the diseased limb, and a Liston's splint to the sound limb; (*b*) subsequently applying a well-fitting Thomas' splint.

*Abscess in the Joint.*—In the hip-joint abscesses are best left alone till all hope of absorption is gone; then you should open them antiseptically, inserting a drainage tube.

*Excision* of the hip-joint is a most unsatisfactory operation, and should only be done as an alternative to amputation through the joint. The reasons why it is so unsatisfactory are (1) the impossibility of thoroughly removing every scrap of diseased tissue; (2) the impossibility of absolutely fixing the joint afterwards.

Now in the knee it is different. Excision is essentially the best treatment when an abscess occurs in the joint, because you can thoroughly carry out the above very necessary conditions. By means of employing steel dowels to fasten the bones together—which may be left in for weeks without causing irritation—and afterwards putting up the limb into P. H. Watson's splint, absolute immobilisation may be secured.

Mr. Lloyd quoted twelve cases of children under twelve whose knees he had excised lately, getting primary union in every case.

